# Effects of Short-Acting Opioids on Intraocular Pressure during General Anesthesia: Systematic Review and Network Meta-Analysis

**DOI:** 10.3390/ph15080989

**Published:** 2022-08-11

**Authors:** Jian-You Huang, Ping-Cheng Shih, Chu-Ting Chen, Han-Yu Lin, Yung-Jiun Chien, Meng-Yu Wu, Chih-Hao Chen, Chun-Yu Chang

**Affiliations:** 1 Department of Anesthesiology, Taipei Tzu Chi Hospital, Buddhist Tzu Chi Medical Foundation, New Taipei City 231, Taiwan; 2 School of Medicine, Tzu Chi University, Hualien 970, Taiwan; 3 Department of Physical Medicine and Rehabilitation, Taipei Tzu Chi Hospital, Buddhist Tzu Chi Medical Foundation, New Taipei City 231, Taiwan; 4 Department of Emergency Medicine, Taipei Tzu Chi Hospital, Buddhist Tzu Chi Medical Foundation, New Taipei City 231, Taiwan; 5 Department of Otolaryngology-Head and Neck Surgery, Taipei Veterans General Hospital, Taipei 112, Taiwan

**Keywords:** endotracheal intubation, intraocular pressure, network meta-analysis, remifentanil, succinylcholine

## Abstract

Intraocular pressure (IOP) is crucial to the well-being of eyes. During anesthesia, the administration of succinylcholine and endotracheal intubation are associated with an increase in IOP, which may be attenuated by short-acting opioids. However, the drug of choice among the commonly used short-acting opioids is unclear. This study aimed to evaluate the effects of fentanyl, sufentanil, alfentanil, and remifentanil on IOP measured after the administration of succinylcholine and after endotracheal intubation in patients undergoing general anesthesia. Five databases were searched. Randomized controlled trials (RCTs) that compared short-acting opioids and reported at least one of the clinical outcomes of interest were included. Nine RCTs with 357 patients were included. Remifentanil (1 μg kg^−1^) more effectively alleviated the increase in IOP than the placebo after the administration of succinylcholine [mean difference (MD) of IOP, −3.64; confidence interval (CI), −5.47 to −1.81 and after endotracheal intubation (MD, −9.71; CI, −11.91 to −7.51). Remifentanil (1 μg kg^−1^) ranked the best in terms of both attenuating the increase in IOP after the administration of succinylcholine [surface under the cumulative ranking curve (SUCRA), 0.91; normalized entropy (NE), 0.47; and after endotracheal intubation (SUCRA, 0.89; NE, 0.54) among all of the treatments. Remifentanil (1 μg kg^−1^) should be considered the drug of choice in the circumstances where increased IOP is a great concern.

## 1. Introduction

Intraocular pressure (IOP) is crucial in determining the ocular perfusion pressure, and is affected by numerous systemic parameters [1]. During anesthesia and surgery, IOP may be increased due to coughing, hypercapnia, and specific surgical procedures that require carbon dioxide pneumoperitoneum and Trendelenburg positioning [2]. An increase in IOP reduces ocular blood flow, leading to optic nerve edema and ischemia, and may result in uncommon but cataclysmic postoperative visual loss [3]. A rapid sequence induction technique may be adopted for emergency surgery to minimize the risk of pulmonary aspiration. In such circumstances, depolarizing neuromuscular blocking agents (e.g., succinylcholine) is still considered the drug of choice for its rapid onset of action [4]. However, succinylcholine has been known to cause an increase in IOP, the use of which in penetrating eye injury is a great concern due to the possible expulsion of ocular contents [5]. This may be further aggravated by endotracheal intubation.

Several anesthetic techniques have been demonstrated to effectively attenuate the increase in IOP after the administration of succinylcholine and endotracheal intubation, and during anesthetic maintenance [2,6]. Short-acting opioids (e.g., fentanyl, alfentanil, sufentanil, and remifentanil) are commonly used perioperatively and provide effective analgesia to surgical stimuli. During laryngoscopy and endotracheal intubation, short-acting opioids effectively suppress airway reflexes and blunt sympathetic nervous responses that would otherwise lead to hemodynamic instability [7,8,9]. Moreover, it has been well-demonstrated that remifentanil and alfentanil effectively attenuated the increase in IOP after intubation, resulting in significantly lower IOP than the placebo [10,11,12]. In the study by Ng et al., they concluded that remifentanil, but not fentanyl, could obtain the increase in IOP associated with succinylcholine and endotracheal intubation [13]. In contrast, both remifentanil and fentanyl were shown to have similar effects on IOP in another study [14]. It remains unclear which a short-acting opioid is the drug of choice to best alleviate the increase in IOP after the administration of succinylcholine and endotracheal intubation. This systematic review and network meta-analysis was therefore conducted in the hope of providing further evidence for clinical practice.

## 2. Methods

### 2.1. Study Design

This systematic review and network meta-analysis aimed to evaluate the effects of four short-acting opioids (i.e., fentanyl, sufentanil, alfentanil, and remifentanil) on intraocular pressure during general anesthesia. The primary outcome is the intraocular pressure after endotracheal intubation. The secondary outcome is the intraocular pressure after the administration of succinylcholine. The present review has been registered with The International Prospective Register of Systematic Reviews (PROSPERO registration number CRD42021256124), and complies with the Preferred Reporting Items for Systematic Review and Meta-analyses (PRISMA) extension statement for network meta-analyses [15].

### 2.2. Search Strategy

Two authors (J.-Y.H. and P.-C.S.) searched PubMed, Embase, Scopus, Web of Science, and Cochrane Library from the earliest available date in each database through 4 April 2021. Subject headings (i.e., MeSH terms in PubMed and Cochrane Library, and Emtree terms in Embase) and search field tags of title, abstracts, and keywords were used to facilitate searching. The following terms were used to search for relevant records: “remifentanil”, “ultiva”, “fentanyl”, “phentanyl”, “fentanest”, “sublimaze”, “duragesic”, “fentora”, “sufentanil”, “sulfentanyl”, “sulfentanil”, “sufenta”, “alfentanil”, “alfentanyl”, “alfenta”, “limifen”, “rapifen”, “fanaxal”, “intraocular pressure”, and “ocular perfusion pressure”. The search queries were constructed by using the Boolean operator “AND” and “OR” to intersect different and cover similar concepts, respectively. The identified records were screened by titles, abstracts, and keywords. Studies with potential eligibility were then subject to full-text review. The reference lists of the included studies were manually searched to identify additional studies. The detailed search queries are available in Supplementary Table S1.

### 2.3. Eligibility Criteria

All studies were assessed for eligibility by two authors (J.-Y.H. and P.-C.S.) according to the following criteria, with all conditions being met: (a) the study consisted of a randomized controlled trial that compared different short-acting opioids (i.e., fentanyl, sufentanil, alfentanil, and remifentanil) in patients who had undergone surgery that required general anesthesia; (b) the study reported at least one of the clinical outcomes of interest including the IOP measured after endotracheal intubation and that measured after the administration of succinylcholine; (c) the full paper of the study could be obtained. Studies were excluded if they were disconnected from the network map. A third author (C.-Y.C.) provided a consensus or discussion if there was any discrepancy in the study selection.

### 2.4. Risk of Bias Assessment

The methodological quality of randomized controlled trials was assessed using the revised Cochrane Risk of Bias Tool [16]. Disagreements in the assessment were resolved through consensus or discussion.

### 2.5. Data Extraction

Datasets were extracted by two authors (J.-Y.H. and C.-Y.C.) from each eligible study. The required information included the author’s name, publication year, number of patients, surgery that the patients received, anesthetic regimen, intervention arms, and effect estimates for the clinical outcomes of interest. In studies in which the outcomes of interest were reported as graphical results, the numerical data were extracted with WebPlotDigitizer Software [17]. The reliability of WebPlotDigitizer has previously been validated [18], and cited in a peer-reviewed article [6].

### 2.6. Statistical Analysis

Estimates for the relative treatment effects of the competing interventions were the mean difference (MD) for continuous outcomes. In studies in which the continuous outcomes were presented as medians and interquartile ranges, the means and standard deviations were estimated using Wan’s method [19,20]. Pairwise meta-analyses were performed to compare different treatment arms directly. Under the assumption of consistency and transitivity, frequentist network meta-analyses were performed for each outcome using the contrast-based fixed effect model to combine the direct and indirect evidence [21]. We estimated the probabilities of each treatment being at each rank, and obtained a treatment ranking using the surface under the cumulative ranking (SUCRA) curve [22]. The normalized entropy (NE) was then calculated to measure the uncertainty of treatment ranking for each treatment. In brief, NE ranges from 0 to 1, with 0 indicating the least uncertain ranking and 1 indicating the most uncertain ranking. Although no definite threshold was defined to indicate a considerable ranking uncertainty, some suggested dividing the NE into 4 groups, i.e., perfect (0–0.2), high (0.2–0.4), medium (0.4–0.6), and low (more than 0.6) [23]. We evaluated the potential inconsistency by using the design-by-treatment interaction model [24], loop inconsistency model [24], and node-splitting model [25]. The comparison-adjusted funnel plot and Egger’s test were used to assess the publication bias [26]. A *p*-value < 0.05 was considered as statistically significant. All statistical analyses were performed using statistical software package Stata, version 15 (StataCorp, College Station, TX, USA).

## 3. Results

### 3.1. Study Selection

**Figure 1:**
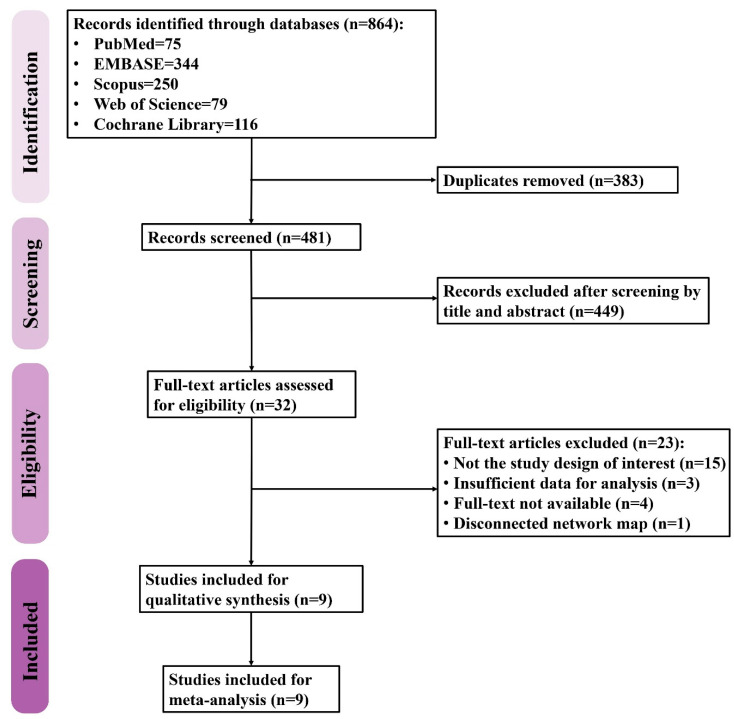
The flow diagram of study selection.

The PRISMA flow diagram of study selection is shown in Figure 1. A total of 864 records were retrieved from five databases including PubMed (n = 75), Embase (n = 344), Scopus (n = 250), Web of Science (n = 79), and Cochrane Library (n = 116). After removing the duplicates, 481 records were screened for eligibility, 32 of which were then assessed with a full-text review while the rest were excluded due to irrelevance. Twenty-three studies were thereafter excluded for being not the study design of interest (n = 15) due to the insufficient data for analysis (n = 3), being unavailable for full-text review (n = 4), and resulting in a disconnected network map (n = 1). Finally, a total of nine studies were included in the present study.

### 3.2. Study Characteristics and Risk of Bias

The study characteristics are shown in Table 1. All of the included studies were randomized controlled trials. Four studies enrolled patients undergoing ophthalmic surgery [10,27,28,29], four studies enrolled patients undergoing non-ophthalmic surgery [11,12,14,30], and one study enrolled patients undergoing elective surgery without specifying it [13]. In studies in which the patient underwent ophthalmic surgery, the IOP was measured on the non-operated eye. In contrast, the IOP was measured on either one of the eyes or both in studies in which the patient underwent non-ophthalmic surgery. Three studies compared alfentanil to placebo [11,12,28], two studies compared remifentanil to alfentanil [27,30], one study compared remifentanil to fentanyl [14], one study compared remifentanil to the placebo [10], one study compared fentanyl to alfentanil [29], and one study compared remifentanil, fentanyl, and the placebo [13]. For endotracheal intubation, succinylcholine was used in six studies [10,11,12,13,27,29], vecuronium was used in two studies [14,30], and both succinylcholine and vecuronium were used in one study [28]. The study drugs were administered before hypnotics in five studies [13,14,27,28,29], between hypnotics and neuromuscular blocking agents in two studies [11,12], and after both hypnotics and neuromuscular blocking agents in two studies [10,30]. Endotracheal intubation was performed following the administration of neuromuscular blocking agents in all studies. The dosage of the short-acting opioids and the anesthesia regimen in each study are presented in detail in Table 1. The assessment of the risk of bias for each included study is presented in Supplementary Figure S1.

### 3.3. IOP after Endotracheal Intubation

**Figure 2:**
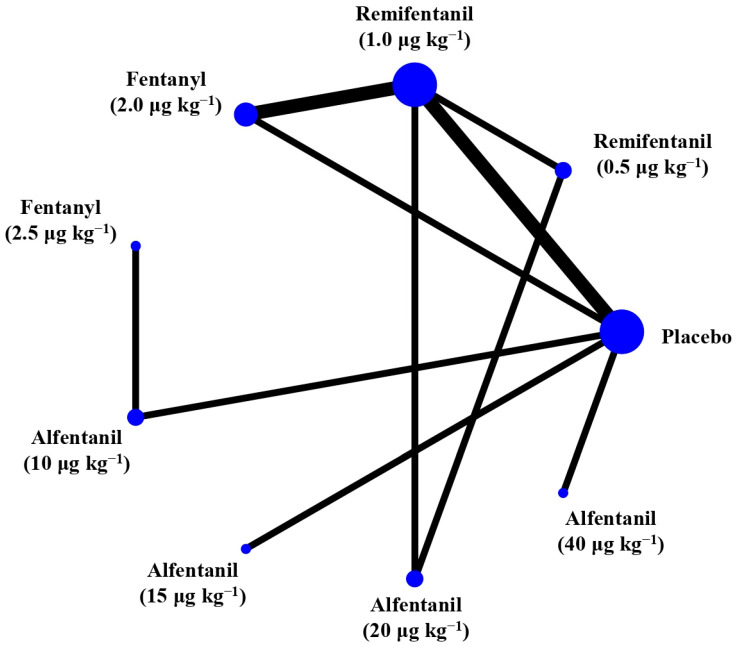
The network treatment comparisons for studies investigating the effects of short-acting opioids on intraocular pressure measured after endotracheal intubation. Each node represents a treatment, and the size of which corresponds to the number of patients studied with each treatment. Treatments that were compared directly are joined with a line, the thickness of which corresponded to the number of trials that assessed the comparisons.

**Figure 3:**
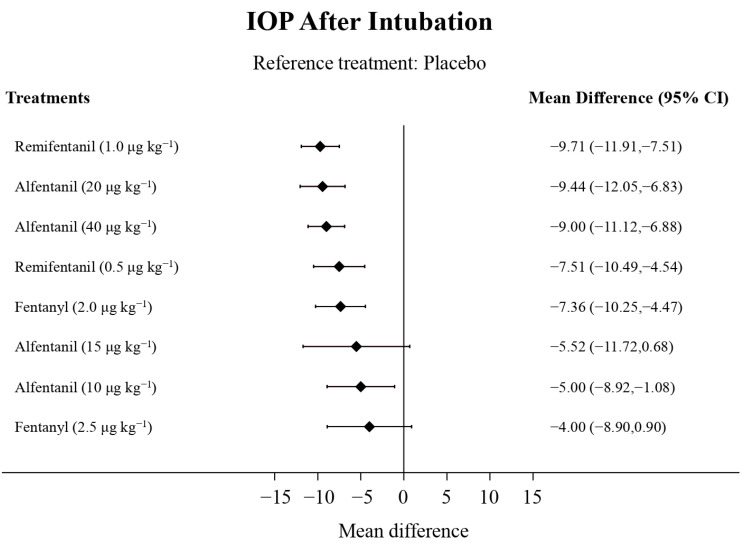
The forest plot depicts the effects of each treatment on the intraocular pressure measured after endotracheal intubation relative to the placebo.

**Figure 4:**
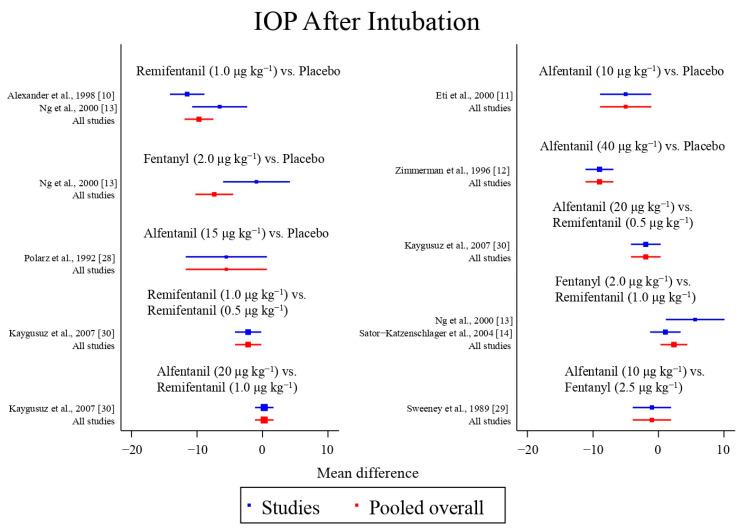
The network meta-analysis forest plot. The blue dots and lines indicate the studies contributing the direct evidence. The red dots and lines indicate the pooled overall network estimates of the treatment effects. See refs [10,11,12,13,14,28,29,30].

**Figure 5:**
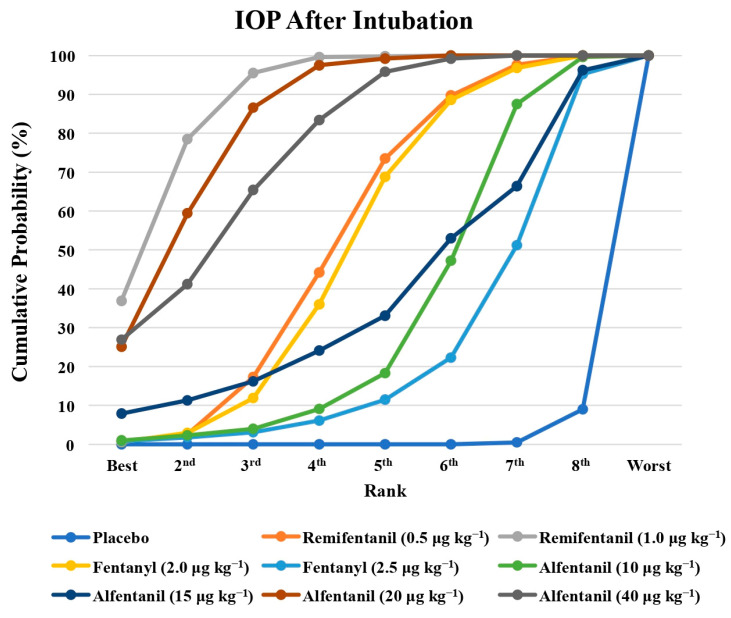
The cumulative ranking probability of the effects of each treatment on the intraocular pressure after endotracheal intubation.

Eight studies reported the IOP measured after endotracheal intubation, and were included in the pairwise meta-analysis. Overall, 10 different comparisons were conducted, and most of them were performed in a single study, with the exception of two pairs (Supplementary Figure S2). Network meta-analysis was conducted and consisted of eight active treatment agents and one placebo (Figure 2). The effects of each treatment on IOP relative to the placebo are shown in Figure 3, and the relative effects of all the competing treatments are summarized in Table 2. Direct comparisons are displayed along with the pooled overall treatment effects in the network meta-analysis forest plot (Figure 4). The cumulative ranking probability of each treatment is shown in Figure 5. The SUCRA and NE values of each treatment are presented in Table 3. Remifentanil at the dose of 1.0 μg kg^−1^ ranked highly with a SUCRA of 0.89 and a NE of 0.54, followed by alfentanil at the doses of 20 μg kg^−1^ (SUCRA, 0.84; NE, 0.65) and 40 μg kg^−1^ (SUCRA, 0.77; NE, 0.77). Fentanyl and a lower dose of remifentanil and alfentanil appeared to be less effective in attenuating the increase in intraocular pressure after endotracheal intubation.

Comparisons between treatments should be read from left to right, and the estimate is in the cell in common between the column-defining treatment and the row-defining treatment. The network estimates from the network meta-analysis are in the lower triangle, and the direct treatment estimates from the pairwise comparisons are in the upper triangle. The estimates are presented as the mean difference (95% confidence interval).

### 3.4. IOP after the Administration of Succinylcholine

**Figure 6:**
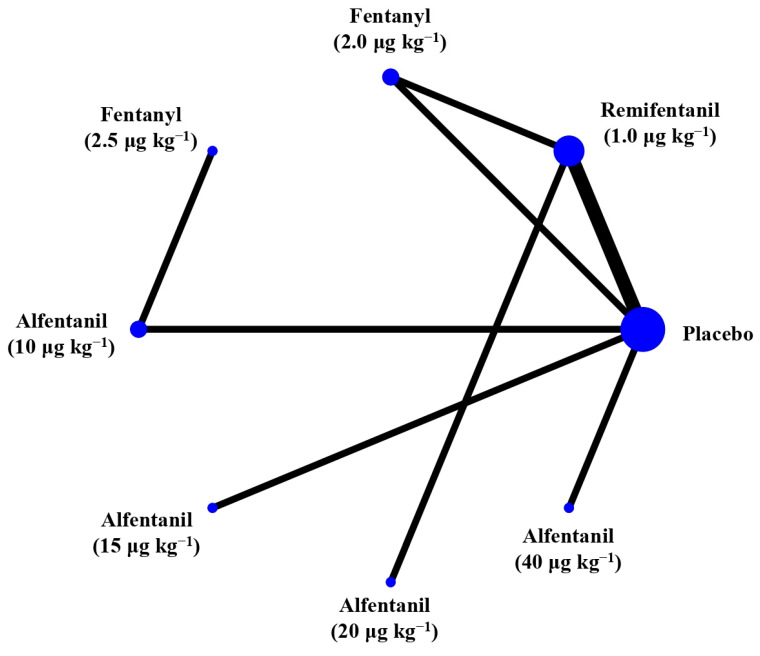
The network treatment comparisons for studies investigating the effects of short-acting opioids on intraocular pressure measured after succinylcholine. Each node represents a treatment, the size of which corresponds to the number of patients studied with each treatment. Treatments that were compared directly are joined with a line, the thickness of which corresponded to the number of trials that assessed the comparisons.

**Figure 7:**
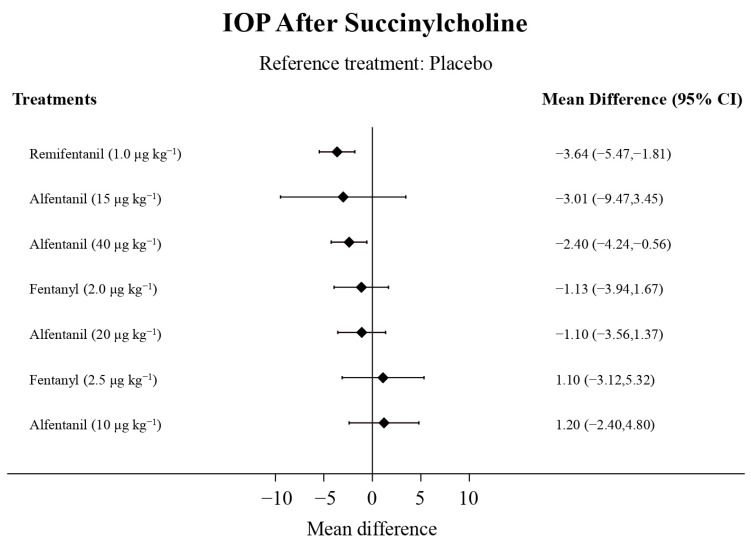
The forest plot depicts the effects of each treatment on the intraocular pressure measured after succinylcholine relative to the placebo.

**Figure 8:**
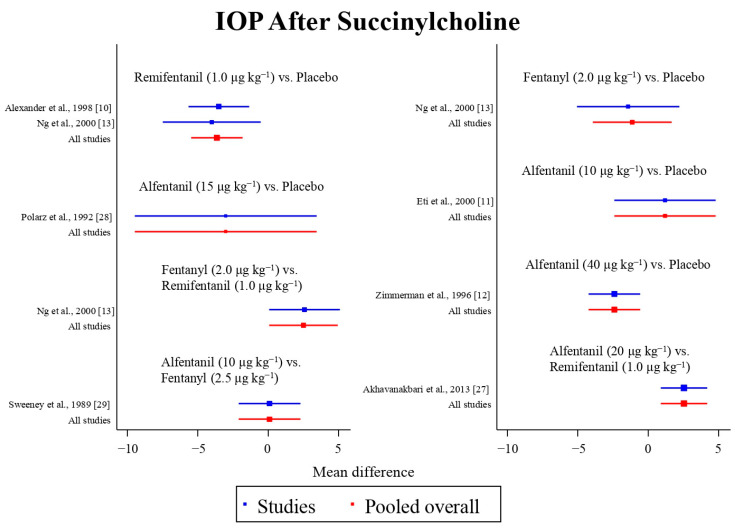
The network meta-analysis forest plot. The blue dots and lines indicate the studies contributing the direct evidence. The red dots and lines indicate the pooled overall network estimates of the treatment effects. See refs [10,11,12,13,27,28,29].

**Figure 9:**
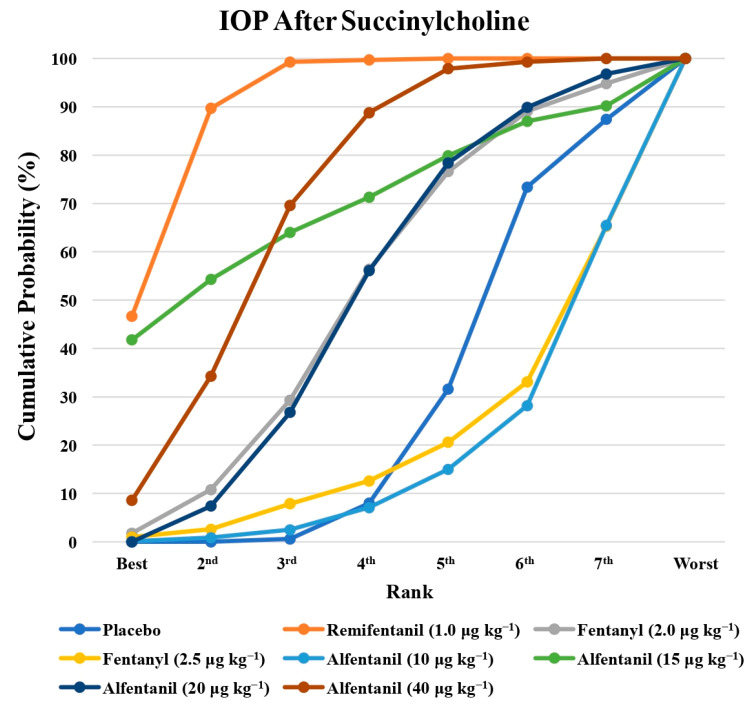
The cumulative ranking probability of the effects of each treatment on the intraocular pressure after succinylcholine.

Eight studies reported the IOP measured after the administration of succinylcholine, and were included in the pairwise meta-analysis. Overall, eight different comparisons were conducted, and most of them were performed in a single study, with the exception of one pair (Supplementary Figure S3). Network meta-analysis was conducted and consisted of seven active treatment agents and one placebo (Figure 6). The effects of each treatment on IOP relative to the placebo are shown in Figure 7, and the relative effects of all of the competing treatments are summarized in Table 4. Direct comparisons are displayed along with the pooled overall treatment effects in the network meta-analysis forest plot (Figure 8). The cumulative ranking probability of each treatment is shown in Figure 9. The SUCRA and NE values of each treatment are presented in Table 3. Remifentanil at the dose of 1.0 μg kg^−1^ ranked highly with a SUCRA of 0.91 and a NE of 0.47. Other short-acting opioids including fentanyl and alfentanil appear to be less effective in attenuating the increase in intraocular pressure after the administration of succinylcholine.

The comparisons between treatments should be read from left to right, and the estimate is in the cell in common between the column-defining treatment and the row-defining treatment. The network estimates from network meta-analysis are in the lower triangle, and the direct treatment estimates from pairwise comparisons are in the upper triangle. Estimates are presented as the mean difference (95% confidence interval).

### 3.5. Inconsistency

In terms of the IOP measured after endotracheal intubation, global inconsistency was detected by the design-by-treatment interaction model (*p* = 0.011) and the loop inconsistency model (*p* = 0.022), which was primarily attributed to the significant difference in the effect among remifentanil at the dose of 1.0 μg kg^−1^, fentanyl at the dose of 2.0 μg kg^−1^, and placebo in the study by Ng et al. [13], Alexander et al. [10], and Sator-Katzenschlager et al. [14], respectively. Inconsistency between the direct and indirect comparisons of fentanyl at the dose of 2.0 μg kg^−1^ and placebo was also observed in the side-splitting models (*p* = 0.003). In terms of the IOP measured after the administration of succinylcholine, no global inconsistency and inconsistency between the direct and indirect comparisons were observed.

### 3.6. Publication Bias

The comparison-adjusted funnel plots for both outcomes are presented in Supplementary Figure S4. Egger’s test revealed no significant publication bias for both outcomes (*p* = 0.428 and *p* = 0.402 for the IOP measured after endotracheal intubation and the administration of succinylcholine, respectively).

## 4. Discussion

The principle finding of the present study is that remifentanil at the dose of 1.0 μg kg^−1^ best alleviated the increase in IOP after endotracheal intubation, which was followed by alfentanil at a higher dose (20 μg kg^−1^ and 40 μg kg^−1^). In contrast, fentanyl, a lower dose of remifentanil and alfentanil, and placebo had lower rankings. The relatively lower NE value of remifentanil at the dose of 1.0 μg kg^−1^ indicates that the SUCRA ranking is less uncertain than the others. In terms of the IOP measured after the administration of succinylcholine, remifentanil at the dose of 1.0 μg kg^−1^ ranked the highest, and the relatively lower NE value indicates that the SUCRA ranking was less uncertain.

The mechanism underlying the succinylcholine-induced increase in IOP is not clearly understood. It may involve the fasciculation of extraocular muscles and transient dilatation of the choroidal blood vessels [31]. Another explanation is the cycloplegic action of succinylcholine, which flattens the lens, and increases the anterior chamber size and outflow resistance due to decreased tension on the scleral spur [32]. Endotracheal intubation is a potent stimulus and is associated with increased plasma catecholamine concentrations, blood pressure, ocular blood flow, and IOP [33,34]. In contrast, opioids possess an IOP-lowering property that may counteract the effects of succinylcholine and endotracheal intubation. The mechanism by which opioids reduce the IOP involves the central diencephalic control centers through relaxation of the ocular muscles as well as the facilitation and inhibition of aqueous humor drainage and production. They may also affect IOP through their effects on the hemodynamic system indirectly [35].

Remifentanil is a fentanyl derivative in the phenylpiperidine family of opioid agents. The p*K*a of remifentanil is 7.1 to 7.2, which is lower than the physiological pH. As a result, a higher proportion of remifentanil molecules are present in their non-ionized form after entering the circulation. This lipid-soluble non-ionized form of remifentanil molecules quickly penetrates the blood–brain barrier, leading to a faster equilibration across the plasma and effect site [36]. The onset of the clinical effects of remifentanil is approximately 1.5 min [37]. Similarly, the p*K*a of alfentanil is 6.5, and at physiological pH, alfentanil molecules are present more in their non-ionized form. This accounts for the fast onset of clinical effects within 2 min [38]. In contrast, fentanyl has a p*K*a of 8.4 with less than 10% of the non-ionized form at the physiological pH. Consequently, the onset of the clinical effects of fentanyl occurs approximately 3 to 5 min after the administration, which is slower than that of remifentanil and alfentanil [36]. The difference in the physicochemical and pharmacokinetic properties of these short-acting opioids may in part explain the findings of our study. The onset of remifentanil coincides with that of succinylcholine, which is approximately 30 to 60 s after intravenous administration. As a result, remifentanil may be superior to alfentanil and fentanyl to attenuate the increase in IOP after the administration of succinylcholine. Under normal circumstances, endotracheal intubation is generally performed after the patients have been completely paralyzed. The time elapse between the administration of either succinylcholine or other non-depolarizing neuromuscular blocking agents and endotracheal intubation allows for the effects of alfentanil to develop. This is consistent with our findings that both remifentanil and alfentanil are superior to fentanyl in terms of effective attenuation of the increase in IOP after intubation.

Study drugs were diluted to 5 mL in two studies [10,28] and 10 mL in one study [13]. All study drugs were given as a bolus, with some of which given over 30 s [13,14,27]. In most of the studies, the heart rate and blood pressure decreased significantly compared with the baseline values after the administration of test drugs and induction agents. Remifentanil is known to cause dose-dependent hypotension and bradycardia, and is recommended to be administered over a period of 30 to 60 s in non-intubated patients. In the included studies, no incidence of bradycardia or hypotension was reported after the administration of test drugs.

Inconsistency is observed in the IOP measured after endotracheal intubation, and is attributed to the significant difference in three of the included studies. The mean difference in IOP measured after endotracheal intubation between remifentanil at the dose of 1.0 μg kg^−1^ and the placebo was greater in the study by Alexander et al. [10] (12.7 mmHg vs. 24.2 mmHg) than in the study by Ng et al. [13] (18.5 mmHg vs. 25.1 mmHg). In addition, the effect of fentanyl at the dose of 2.0 μg kg^−1^ (24.1 mmHg) on the IOP measured after endotracheal intubation was similar to that of the placebo (25.1 mmHg), and was less effective than remifentanil at the dose of 1.0 μg kg^−1^ (18.5 mmHg) in the study by Ng et al. [13]. However, although fentanyl at the dose of 2.0 μg kg^−1^ was also less effective than remifentanil at the dose of 1.0 μg kg^−1^, the mean difference was smaller in the study by Sator-Katzenschlager et al. (8.9 mmHg vs. 7.8 mmHg) [14]. The reason underlying the inconsistency observed is unclear. The possible explanation may be the difference in the preparation and administration of the study drugs. In the study by Ng et al., the study drugs were diluted to 10 mL and administered as a bolus over 30 s. In contrast, in the study by Alexander et al., the study drugs were diluted to 5 mL. In the study by Sator-Katzenschlager et al., fentanyl was administered without dilution.

There were some limitations in the present study. First, only nine studies met the inclusion criteria, and most of the direct comparison was contributed by a single trial. Second, although the effects of sufentanil and fentanyl on the IOP measured after endotracheal intubation were investigated by Stirt et al., the inclusion of this would have led to a disconnected network map and prevented further analysis. As a result, it was not included in the present study. Third, although there was a trend that a higher dose of remifentanil and alfentanil more effectively alleviated the increase in IOP measured after endotracheal intubation than the lower dose ones, definitive conclusion cannot be drawn due to a relatively small number of trials. Further investigations are required to confirm this observation. Finally, although network meta-analysis combined both the direct and indirect evidence, and increased the sample size by pooling the multiple study results and patients, it remains difficult to evaluate the power to detect a statistically significant difference. The present study included all the available studies, but the total number of trials was still low. As a result, some of the results may not have sufficient power to detect the difference.

In conclusion, remifentanil at the dose of 1.0 μg kg^−1^ best attenuated the increase in IOP measured after the administration of succinylcholine and endotracheal intubation. Alfentanil at the dose of 20 μg kg^−1^ and 40 μg kg^−1^ may be an alternative drug of choice to remifentanil.

## Figures and Tables

**Table 1:** The study characteristics.

Study	Location	Surgery	Sample Size	Mean Age	Intervention Arm	Anesthesia Regimen
Akhavanakbari et al., 2013 [27]	Iran	Elective cataract surgery	50	69.1	Remifentanil 1.0 μg kg^−1^ Alfentanil 20 μg kg^−1^	Induction: thiopental (5 mg kg^−1^), succinylcholine (1 mg kg^−1^) Maintenance: propofol (100 μg kg^−1^ min^−1^), atracurium (0.1–0.2 mg kg^−1^), remifentanil (0.1 μg kg^−1^ min^−1^) or alfentanil (0.5 μg kg^−1^ min^−1^)
Kaygusuz et al., 2007 [30]	Turkey	Elective non-ophthalmic surgery left	60	30.0	Alfentanil 20 μg kg^−1^ Remifentanil 1.0 μg kg^−1^ Remifentanil 0.5 μg kg^−1^	Premedication: midazolam (0.07 mg kg^−1^) intramuscularly 30 min before induction Induction: propofol (2 mg kg^−1^), vecuronium (0.1 mg kg^−1^), study drugs injected by diluting with 5 mL normal saline immediately following induction agents Maintenance: 2% sevoflurane
Sator-Katzenschlager et al., 2004 [14]	Vienna	Elective non-ophthalmic surgery both	32	53.0	Remifentanil 1.0 μg kg^−1^ Fentanyl 2.0 μg kg^−1^	Premedication: midazolam (7.5 mg) orally 1 h before surgery Induction: propofol (2 mg kg^−1^), vecuronium (0.1 mg kg^−1^) Maintenance: propofol (4–8 mg kg^−1^ h^−1^), vecuronium (0.03 mg kg^−1^), remifentanil (0.25–0.5 μg kg^−1^ min^−1^), fentanyl (2–5 μg kg^−1^ bolus doses as clinically indicated)
Eti et al., 2000 [11]	Turkey	Elective non-ophthalmic surgery right	40	34.6	Propofol 2.5 mg kg^−1^ + succinylcholine 1.5 mg kg^−1^ Propofol 2.5 mg kg^−1^ + alfentanil 10 μg kg^−1^ + succinylcholine 1.5 mg kg^−1^ Thiopental 5.0 mg kg^−1^ + succinylcholine 1.5 mg kg^−1^ Thiopental 5.0 mg kg^−1^ + vecuronium 0.1 mg kg^−1^	Induction regimens have been described in the intervention arm column
Ng et al., 2000 [13]	Singapore	Elective surgery left	45	32.8	Remifentanil 1.0 μg kg^−1^ Fentanyl 2.0 μg kg^−1^ Normal saline	Premedication: amethocaine 1% drops instilled on patient’s left eye Induction: thiopental (5 mg kg^−1^), succinylcholine (2 mg kg^−1^) Maintenance: 1% isoflurane, atracurium
Alexander et al., 1998 [10]	North Carolina, USA	Elective eye surgery	30	59.1	Remifentanil 1.0 μg kg^−1^ Normal saline	Premedication: midazolam (0.03 mg kg^−1^), tetracaine 0.5% drops to non-operated eye Induction: propofol (2 mg kg^−1^), succinylcholine (1 mg kg^−1^) Maintenance: 1% isoflurane
Zimmerman et al., 1996 [12]	California, USA	Elective non-ophthalmic Surgery left	60	30.0	Thiopental 5.0 mg kg^−1^ + succinylcholine 1.5 mg kg^−1^ Propofol 2.0 mg kg^−1^ + succinylcholine 1.5 mg kg^−1^ Propofol 2.0 mg kg^−1^ + alfentanil 40 μg kg^−1^ + succinylcholine 1.5 mg kg^−1^	Premedication: midazolam (1–2 mg, at the discretion of the anesthesia team), proparacaine (0.5%, one drop in patient’s left eye), lidocaine (0.5 mg kg^−1^) Induction regimens have been described in the intervention arm column
Polarz et al., 1992 [28]	Heidelberg, Germany	Ophthalmic surgery	40	73.8	Alfentanil 15 μg kg^−1^ Placebo	Premedication: midazolam (0.06 mg kg^−1^) and atropine (0.01 mg kg^−1^) intramuscularly Induction: vecuronium (0.01 mg kg^−1^), thiopentone (3–4 mg kg^−1^), succinylcholine (1 mg kg^−1^) Maintenance: 0.5–0.8% isoflurane, vecuronium
Sweeney et al., 1989 [29]	Liverpool, England	Routine ophthalmic operations	40	65.9	Fentanyl 2.5 μg kg^−1^ Alfentanil 10 μg kg^−1^	Premedication: 0.4% benoxinate (one or two drops), diazepam (5–10 mg orally two hours before operation) Induction: thiopentone (2–4 mg kg^−1^), succinylcholine 1.5 mg Maintenance: 0.8% enflurane, atracurium (0.6 mg kg^−1^)

**Table 2:** The comparative efficacy of treatments on the intraocular pressure measured after endotracheal intubation.

Placebo		10.10 (7.87, 12.33)	0.93 (−4.18, 6.03)		5.00 (1.08, 8.92)	5.52 (−0.68, 11.72)		9.00 (6.88, 11.12)
7.51 (4.54, 10.49)	Remifentanil (0.5 μg kg^−1^)	2.20 (0.20, 4.20)					1.93 (−0.33, 4.19)	
9.71 (7.51, 11.91)	2.20 (0.20, 4.20)	Remifentanil (1.0 μg kg^−1^)	−2.03 (−4.10, 0.04)				−0.27 (−1.67, 1.13)	
7.36 (4.47, 10.25)	−0.15 (−3.02, 2.71)	−2.35 (−4.41, −0.29)	Fentanyl (2.0 μg kg^−1^)					
4.00 (−0.90, 8.90)	−3.51 (−9.25, 2.22)	−5.71 (−11.08, −0.34)	−3.36 (−9.05, 2.33)	Fentanyl (2.5 μg kg^−1^)	1.00 (−1.94, 3.94)			
5.00 (1.08, 8.92)	−2.51 (−7.43, 2.41)	−4.71 (−9.20, −0.21)	−2.36 (−7.23, 2.51)	1.00 (−1.94, 3.94)	Alfentanil (10 μg kg^−1^)			
5.52 (−0.68, 11.72)	−1.99 (−8.87, 4.89)	−4.19 (−10.77, 2.39)	−1.84 (−8.68, 5.00)	1.52 (−6.38, 9.42)	0.52 (−6.81, 7.86)	Alfentanil (15 μg kg^−1^)		
9.44 (6.83, 12.05)	1.93 (−0.33, 4.19)	−0.27 (−1.67, 1.13)	2.08 (−0.40, 4.57)	5.44 (−0.11, 10.99)	4.44 (−0.27, 9.15)	3.92 (−2.81, 10.65)	Alfentanil (20 μg kg^−1^)	
9.00 (6.88, 11.12)	1.49 (−2.17, 5.14)	−0.71 (−3.76, 2.35)	1.64 (−1.94, 5.22)	5.00 (−0.34, 10.34)	4.00 (−0.45, 8.45)	3.48 (−3.07, 10.03)	−0.44 (−3.80, 2.92)	Alfentanil (40 μg kg^−1^)

**Table 3:** The surface under the cumulative ranking curve (SUCRA) and normalized entropy (NE) of each treatment.

Intraocular Pressure Measured after Endotracheal Intubation		
Treatments	SUCRA	NE
Remifentanil (1.0 μg kg^−1^)	0.89	0.54
Alfentanil (20 μg kg^−1^)	0.84	0.65
Alfentanil (40 μg kg^−1^)	0.77	0.77
Remifentanil (0.5 μg kg^−1^)	0.53	0.77
Fentanyl (2.0 μg kg^−1^)	0.51	0.76
Alfentanil (15 μg kg^−1^)	0.39	0.89
Alfentanil (10 μg kg^−1^)	0.34	0.70
Fentanyl (2.5 μg kg^−1^)	0.24	0.69
Placebo	0.01	0.15
Intraocular Pressure Measured after the Administration of Succinylcholine		
Treatments	SUCRA	NE
Remifentanil (1.0 μg kg^−1^)	0.91	0.47
Alfentanil (40 μg kg^−1^)	0.71	0.75
Alfentanil (15 μg kg^−1^)	0.70	0.86
Fentanyl (2.0 μg kg^−1^)	0.51	0.89
Alfentanil (20 μg kg^−1^)	0.51	0.84
Placebo	0.29	0.70
Fentanyl (2.5 μg kg^−1^)	0.20	0.77
Alfentanil (10 μg kg^−1^)	0.17	0.70

NE—normalized entropy; SUCRA—the surface under the cumulative ranking curve.

**Table 4:** The comparative efficacy of treatments on the intraocular pressure measured after the administration of succinylcholine.

Placebo	3.64 (1.81, 5.47)	1.42 (−2.22, 5.06)		−1.20 (−4.80, 2.40)	3.01 (−3.45, 9.47)		2.40 (0.56, 4.24)
3.64 (1.81, 5.47)	Remifentanil (1.0 μg kg^−1^)	−2.58 (−5.10, −0.07)				−2.54 (−4.19, −0.90)	
1.13 (−1.67, 3.94)	−2.51 (−4.94, −0.07)	Fentanyl (2.0 μg kg^−1^)					
−1.10 (−5.32, 3.12)	−4.74 (−9.34, −0.14)	−2.23 (−7.30, 2.83)	Fentanyl (2.5 μg kg^−1^)	−0.10 (−2.30, 2.10)			
−1.20 (−4.80, 2.40)	−4.84 (−8.88, −0.80)	−2.33 (−6.89, 2.23)	−0.10 (−2.30, 2.10)	Alfentanil (10 μg kg^−1^)			
3.01 (−3.45, 9.47)	−0.63 (−7.34, 6.08)	1.88 (−5.16, 8.91)	4.11 (−3.60, 11.82)	4.21 (−3.18, 11.60)	Alfentanil (15 μg kg^−1^)		
1.10 (−1.37, 3.56)	−2.54 (−4.19, −0.90)	−0.04 (−2.98, 2.90)	2.20 (−2.69, 7.08)	2.30 (−2.07, 6.66)	−1.91 (−8.83, 5.00)	Alfentanil (20 μg kg^−1^)	
2.40 (0.56, 4.24)	−1.24 (−3.83, 1.35)	1.27 (−2.08, 4.62)	3.50 (−1.10, 8.10)	3.60 (−0.44, 7.64)	−0.61 (−7.32, 6.10)	1.30 (−1.77, 4.38)	Alfentanil (40 μg kg^−1^)

## Data Availability

The data that supports the findings of this study are available from the corresponding author, upon reasonable request.

## Supplementary Materials

The following supporting information can be downloaded at: https://www.mdpi.com/article/10.3390/ph15080989/s1.
