# Supplementary material for: Effects of Short-Acting Opioids on Intraocular Pressure during General Anesthesia: Systematic Review and Network Meta-Analysis
Source: Pharmaceuticals (Basel). 2022 Aug 11;15(8):989. doi: 10.3390/ph15080989 (PMC9412988; doi:10.3390/ph15080989)
Supplement: Supplementary file 1 [file pharmaceuticals-15-00989-s001.zip › pharmaceuticals-1798098-supplementary_revised_CYC.pdf]

Supplementary Materials

Effects of short-acting opioids on intraocular pressure during general anesthesia: Systematic review and network meta-analysis

Jian-You Huang, Ping-Cheng Shih, Chu-Ting Chen, Han-Yu Lin, Yung-Jiun Chien, Meng-Yu Wu, Chih-Hao Chen, Chun-Yu Chang

Supplementary Table S1. Detailed search queries

Database	Query	Results
PubMed	("Remifentanil"[MeSH Terms] OR "remifentanil/administration and dosage"[MeSH Terms] OR "remifentanil/adverse effects"[MeSH Terms] OR "remifentanil/statistics and numerical data"[MeSH Terms] OR "remifentanil/therapeutic use"[MeSH Terms] OR "remifentanil/toxicity"[MeSH Terms] OR "Remifentanil"[Title/Abstract] OR "ultiva"[Title/Abstract] OR "Remifentanil"[All Fields] OR "ultiva"[All Fields] OR "Fentanyl"[MeSH Terms] OR "fentanyl/administration and dosage"[MeSH Terms] OR "fentanyl/adverse effects"[MeSH Terms] OR "fentanyl/statistics and numerical data"[MeSH Terms] OR "fentanyl/therapeutic use"[MeSH Terms] OR "fentanyl/toxicity"[MeSH Terms] OR "phentanyl"[Title/Abstract] OR "fentanest"[Title/Abstract] OR "sublimaze"[Title/Abstract] OR "duragesic"[Title/Abstract] OR "durogesic"[Title/Abstract] OR "fentora"[Title/Abstract] OR "phentanyl"[All Fields] OR "fentanest"[All Fields] OR "sublimaze"[All Fields] OR "duragesic"[All Fields] OR "durogesic"[All Fields] OR "fentora"[All Fields] OR "Sufentanil"[MeSH Terms] OR "sufentanil/administration and dosage"[MeSH Terms] OR "sufentanil/adverse effects"[MeSH Terms] OR "sufentanil/statistics and numerical data"[MeSH Terms] OR "sufentanil/therapeutic use"[MeSH Terms] OR "sufentanil/toxicity"[MeSH Terms] OR "Sufentanil"[Title/Abstract] OR "sulfentanyl"[Title/Abstract] OR "sulfentanil"[Title/Abstract] OR "sufenta"[Title/Abstract] OR "Sufentanil"[All Fields] OR "sulfentanyl"[All Fields] OR "sulfentanil"[All Fields] OR "sufenta"[All Fields] OR "Alfentanil"[MeSH Terms] OR "alfentanil/administration and dosage"[MeSH Terms] OR "alfentanil/adverse effects"[MeSH Terms] OR "alfentanil/statistics and numerical data"[MeSH Terms] OR "alfentanil/therapeutic use"[MeSH Terms] OR "alfentanil/toxicity"[MeSH Terms] OR "Alfentanil"[Title/Abstract] OR "alfentanyl"[Title/Abstract] OR "alfenta"[Title/Abstract] OR "rapifen"[Title/Abstract] OR "Alfentanil"[All Fields] OR "alfentanyl"[All Fields] OR "alfenta"[All Fields] OR ("Alfentanil"[MeSH Terms] OR "Alfentanil"[All Fields]) OR "rapifen"[All Fields] OR ("Alfentanil"[MeSH Terms] OR "Alfentanil"[All Fields])) AND ("Intraocular Pressure"[MeSH Terms] OR "Intraocular Pressure"[Title/Abstract] OR "intraocular pressures"[Title/Abstract] OR "ocular perfusion pressure"[Title/Abstract] OR "ocular perfusion pressures"[Title/Abstract] OR "Intraocular Pressure"[All Fields] OR "intraocular pressures"[All Fields] OR "ocular perfusion pressure"[All Fields] OR "ocular perfusion pressures"[All Fields])	75
Embase	('remifentanil'/exp OR '3 [4 methoxycarbonyl 4 (n phenylpropionamido) piperidino] propionic acid methyl ester' OR '4 (methoxycarbonyl) 4 [(1 oxopropyl) phenylamino] 1 piperidinepropanoic acid methyl ester' OR 'gi 87084' OR 'gi 87084b' OR 'gi87084' OR 'gi87084b' OR 'remifentanil' OR 'remifentanil hydrochloride' OR 'remifentanyl' OR 'ultiva' OR 'fentanyl'/exp OR '1 (2 phenylethyl) 4 (n phenylpropionamido) piperidine' OR '1 phenethyl 4 (n phenylpropionamido) piperidine' OR '1 phenethyl 4 n propionylanilinopiperidine' OR 'ap 48' OR 'ap48' OR 'duragesic' OR 'duragesic 100' OR 'duragesic 12' OR 'duragesic 25' OR 'duragesic 37' OR 'duragesic 50' OR 'duragesic 75' OR 'duragesic-100' OR 'duragesic-12' OR 'duragesic-25' OR 'duragesic-37' OR	344

	'duragesic-50' OR 'duragesic-75' OR 'durogesic' OR 'durogesic dtrans' OR 'durogesic matrix' OR 'durogesic patch' OR 'durogesic smat' OR 'durogesic transdermal system' OR 'epufen' OR 'fentalis' OR 'fentamat' OR 'fentamyl' OR 'fentanex' OR 'fentanil' OR 'fentanyl' OR 'fentanyl buccal soluble film' OR 'fentanyl hydrochloride' OR 'fentanyl iontophoretic' OR 'fentanyl-100' OR 'fentanyl-12' OR 'fentanyl-25' OR 'fentanyl-37' OR 'fentanyl-50' OR 'fentanyl-62' OR 'fentanyl-75' OR 'fentanyl-87' OR 'fetanex' OR 'fetnanyl' OR 'leptanal' OR 'matrifen' OR 'mezolar matrix' OR 'n (1 phenethyl 4 piperidinyl) n phenylpropionamide' OR 'n (1 phenethyl 4 piperidyl) propionanilide' OR 'n (1 phenethylpiperid 4 yl) propionanilide' OR 'n (1 phenethylpiperidin 4 yl) n phenylpropionamide' OR 'n phenethyl 4 (n propionylanilino) piperidine' OR 'n phenyl n [1 (2 phenylethyl) 4 piperidinyl] propanamide' OR 'n phenyl n [1 (2 phenylethyl) piperidin 4 yl] propanamide' OR 'pecfent' OR 'phentanyl' OR 'r 4263' OR 'r4263' OR 'rapinyl' OR 'recuvyra' OR 'subsys' OR 'tanyl' OR 'tilotrans' OR 'transfenta' OR 'sufentanil'/exp OR '4 (n ethylcarbonylanilino) 4 methoxymethyl 1 (2 thien 2 ylethyl) piperidine' OR 'arx 01' OR 'arx01' OR 'dzuveo' OR 'fentanyl sulfate' OR 'fentanyl sulphate' OR 'fentathianyl' OR 'fentathienyl' OR 'fentatienil' OR 'n [4 (methoxymethyl) 1 [2 (2 thienyl) ethyl] 4 piperidyl] propionanilide' OR 'r 30, 730' OR 'r 30730' OR 'r30, 730' OR 'r30730' OR 'sufentanil' OR 'sufentanyl' OR 'sulfentanil' OR 'sulfentanyl' OR 'zalviso' OR 'alfentanil'/exp OR 'alfenil' OR 'alfenta' OR 'alfentanil' OR 'alfentanil hydrochloride' OR 'alfentanyl' OR 'brevafen' OR 'fanaxal' OR 'fentalim' OR 'limifen' OR 'n [1 [2 (4 ethyl 5 oxo 2 tetrazolin 1 yl) ethyl] 4 (methoxymethyl) 4 piperidyl] propionanilide' OR 'r 39, 209' OR 'r 39209' OR 'rapifen') AND ('intraocular pressure'/exp OR 'eye ball pressure' OR 'eye internal pressure' OR 'eye pressure' OR 'eye tension' OR 'eyeball pressure' OR 'intra ocular pressure' OR 'intraocular pressure' OR 'intraocular tension' OR 'intraorbital pressure' OR 'ocular pressure' OR 'ocular tension' OR 'pressure, intraocular' OR 'ocular perfusion pressure'/exp OR 'eye perfusion pressure' OR 'ocular perfusion pressure' OR 'perfusion pressure of the eye')	
Web of Science	TS=(("remifentanil" OR "ultiva" OR "fentanyl" OR "phentanyl" OR "fentanest" OR "sublimaze" OR "duragesic" OR "durogesic" OR "fentora" OR "sufentanil" OR "sulfentanyl" OR "sulfentanil" OR "sufenta" OR "alfentanil" OR "alfentanyl" OR "alfenta" OR "limifen" OR "rapifen" OR "fanaxal") AND ("intraocular pressure" OR "ocular perfusion pressure"))	79
Scopus	TITLE-ABS-KEY(("remifentanil" OR "ultiva" OR "fentanyl" OR "phentanyl" OR "fentanest" OR "sublimaze" OR "duragesic" OR "durogesic" OR "fentora" OR "sufentanil" OR "sulfentanyl" OR "sulfentanil" OR "sufenta" OR "alfentanil" OR "alfentanyl" OR "alfenta" OR "limifen" OR "rapifen" OR "fanaxal") AND ("intraocular pressure" OR "ocular perfusion pressure"))	250
Cochrane Library	Search Name: opioid and iop<br>Date Run: 04/04/2021 11:10:29<br>Comment:	116

ID	Search	Hits
#1	MeSH descriptor: [Remifentanil] explode all trees	1768
#2	MeSH descriptor: [Remifentanil] explode all trees and with qualifier(s): [administration & dosage - AD]	72
#3	MeSH descriptor: [Remifentanil] explode all trees and with qualifier(s): [adverse effects - AE]	22
#4	MeSH descriptor: [Remifentanil] explode all trees	1768
#5	MeSH descriptor: [Remifentanil] explode all trees and with qualifier(s): [therapeutic use - TU]	24
#6	MeSH descriptor: [Remifentanil] explode all trees and with qualifier(s): [toxicity - TO]	0
#7	remifentanil	4855
#8	ultiva	99
#9	MeSH descriptor: [Fentanyl] explode all trees and with qualifier(s): [administration & dosage - AD]	2473
#10	MeSH descriptor: [Fentanyl] explode all trees	5610
#11	MeSH descriptor: [Fentanyl] explode all trees and with qualifier(s): [adverse effects - AE]	935
#12	MeSH descriptor: [Fentanyl] explode all trees	5610
#13	MeSH descriptor: [Fentanyl] explode all trees and with qualifier(s): [therapeutic use - TU]	893
#14	MeSH descriptor: [Fentanyl] explode all trees and with qualifier(s): [toxicity - TO]	2
#15	fentanyl	14988
#16	phentanyl	29
#17	fentanest	23
#18	sublimaze	12
#19	duragesic	26
#20	durogesic	37
#21	fentora	5
#22	MeSH descriptor: [Sufentanil] explode all trees	939
#23	MeSH descriptor: [Sufentanil] explode all trees and with qualifier(s): [administration & dosage - AD]	393
#24	MeSH descriptor: [Sufentanil] explode all trees and with qualifier(s): [adverse effects - AE]	180
#25	MeSH descriptor: [Sufentanil] explode all trees	939
#26	MeSH descriptor: [Sufentanil] explode all trees and with qualifier(s): [therapeutic use - TU]	144
#27	MeSH descriptor: [Sufentanil] explode all trees and with qualifier(s): [toxicity - TO]	0
#28	sufentanil	2877

#29 sufentanyl 108
#30 sulfentanyl 21
#31 sulfentanil 41
#32 sufenta 35
#33 MeSH descriptor: [Alfentanil] explode all trees 700
#34 MeSH descriptor: [Alfentanil] explode all trees and with qualifier(s): [administration & dosage - AD] 270
#35 MeSH descriptor: [Alfentanil] explode all trees and with qualifier(s): [adverse effects - AE] 82
#36 MeSH descriptor: [Alfentanil] explode all trees and with qualifier(s): [therapeutic use - TU] 94
#37 MeSH descriptor: [Alfentanil] explode all trees and with qualifier(s): [toxicity - TO] 0
#38 alfentanil 1523
#39 alfentanyl67
#40 alfenta 3
#41 limifen 1
#42 rapifen 15
#43 fanaxal 0
#44 {OR #1-#43} 22048
#45 MeSH descriptor: [Intraocular Pressure] explode all trees 3405
#46 "intraocular pressure" 8794
#47 "intraocular pressures" 394
#48 "ocular perfusion pressure" 146
#49 "ocular perfusion pressures" 18
#50 {OR #45-#49} 8863
#51 #44 AND #50 121

Trials: 116
Cochrane Reviews: 5

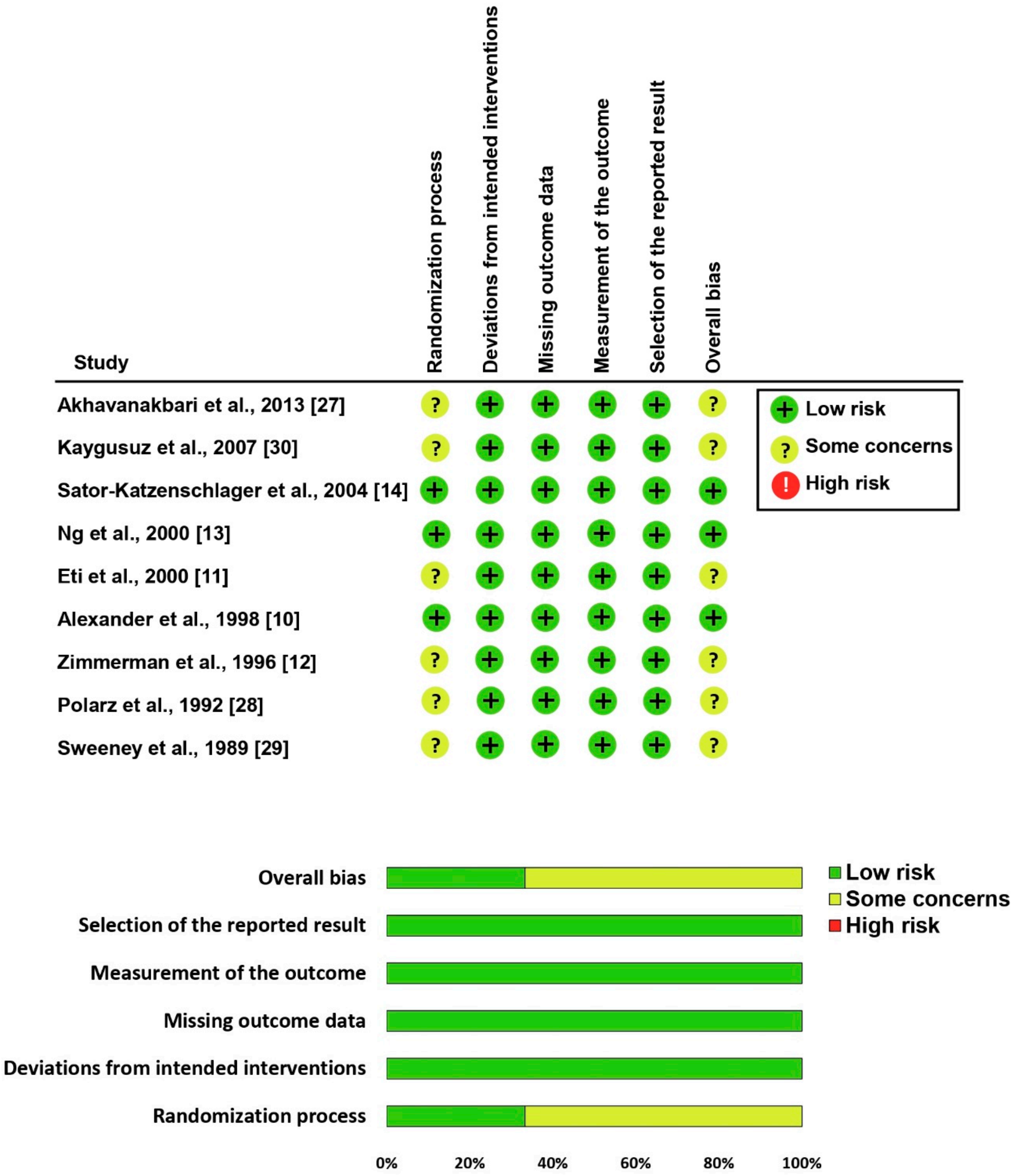

Supplementary Figure S1. Risk of bias summary and graph.

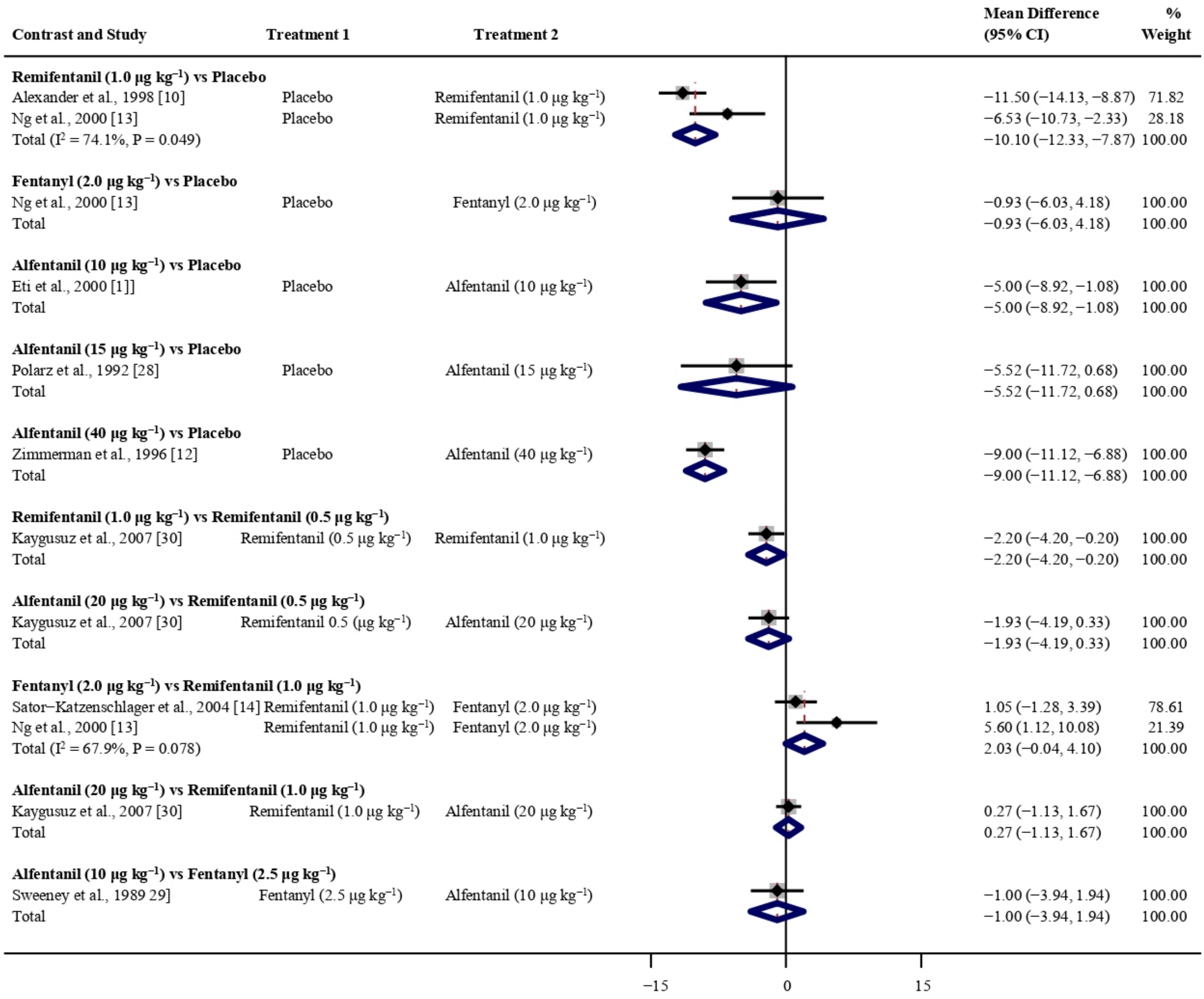

Supplementary Figure S2. Forest plots from pairwise comparisons of treatment agents on intraocular pressure measured after endotracheal intubation.

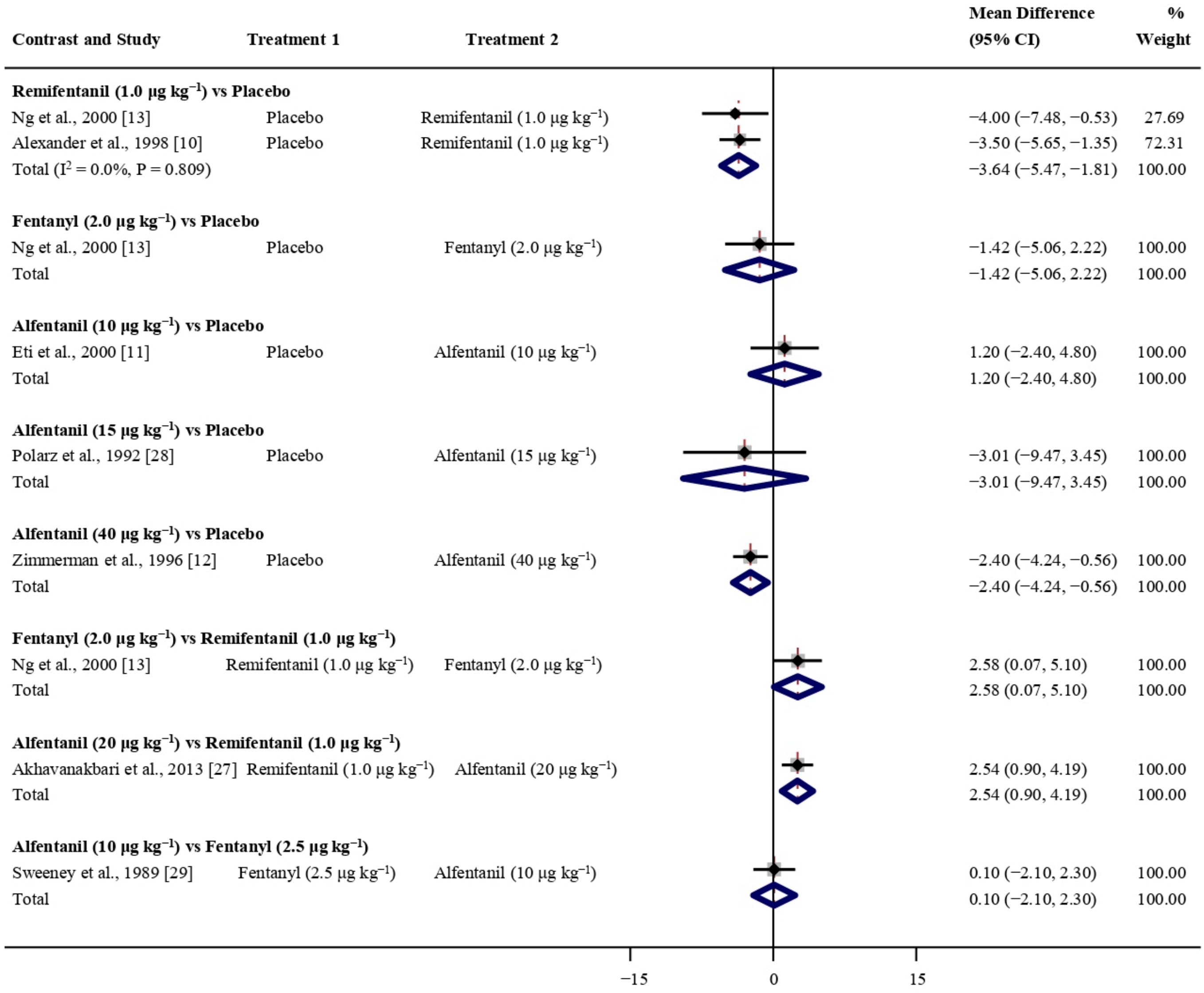

Supplementary Figure S3. Forest plots from pairwise comparisons of treatment agents on intraocular pressure measured after the administration of succinylcholine.

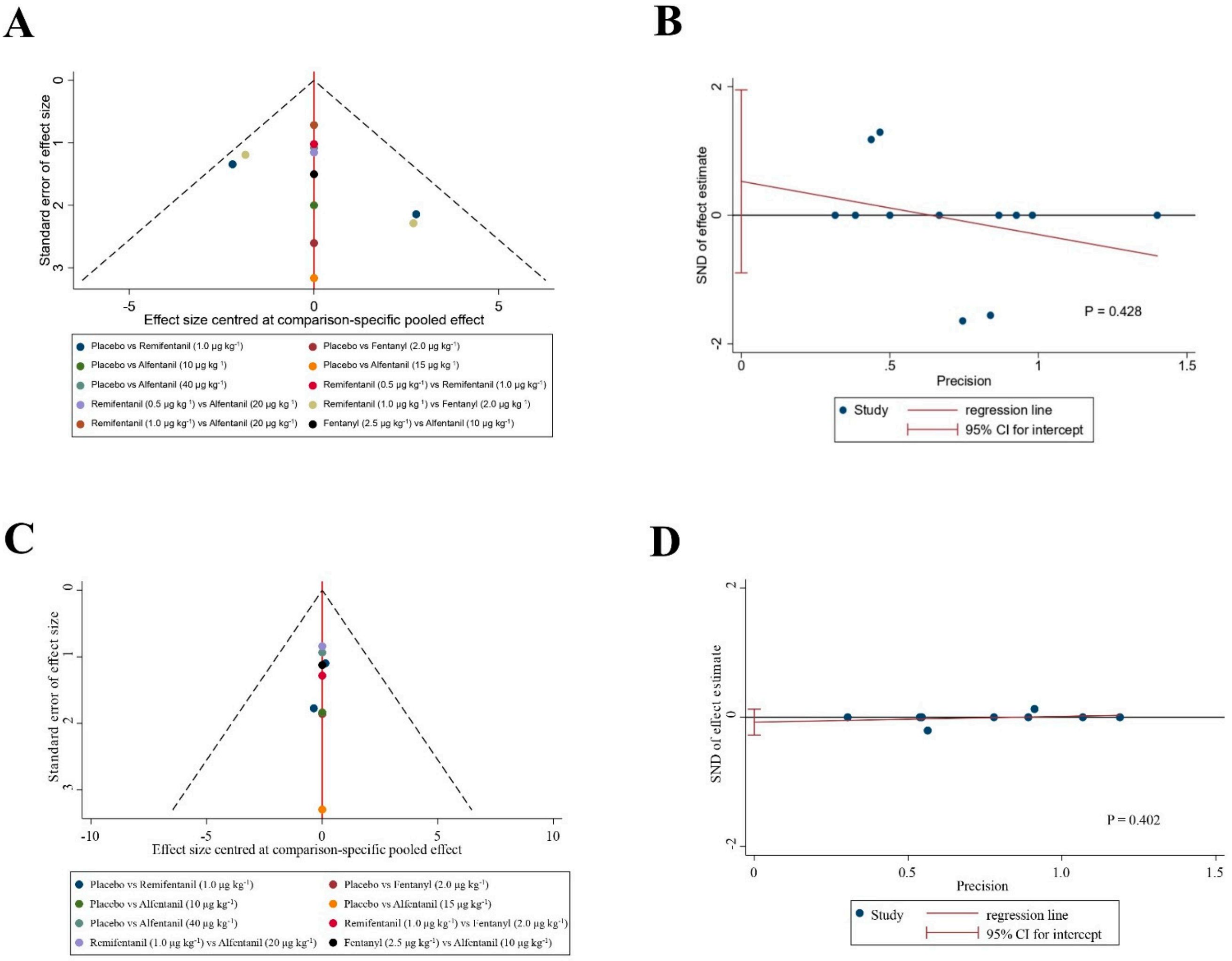

Supplementary Figure S4. (A) Comparison-adjusted funnel plot, and (B) Egger's test for the intraocular pressure measured after endotracheal intubation. (C) Comparison-adjusted funnel plot, and (D) Egger's test for the intraocular pressure measured after the administration of succinylcholine.
